# The Remarkable Genetics of Helicobacter pylori

**DOI:** 10.1128/mbio.02158-22

**Published:** 2022-10-26

**Authors:** Daniel Falush

**Affiliations:** a The Center for Microbes, Development and Health, Key Laboratory of Molecular Virology and Immunology, Institut Pasteur of Shanghai, Chinese Academy of Sciences, Shanghai, People’s Republic of China

**Keywords:** *Helicobacter pylori*, linkage disequilibrium, homologous recombination

## Abstract

The Helicobacter pylori genome is more thoroughly mixed by homologous recombination than by any other organism that has been investigated, leading to apparent “free recombination” within populations. A recent *mBio* article by F. Ailloud, I. Estibariz, G. Pfaffinger, and S. Suerbaum (mBio 13:e01811-22, 2022, https://doi.org/10.1128/mbio.01811-22) helps to elucidate the cellular machinery that is used to achieve these unusual rates of genetic exchange. Specifically, they show that the UvrC gene, which is part of the repair machinery for DNA damage caused by ultraviolet light, has evolved an additional function in *H. pylori*, allowing very short tracts of DNA—with a mean length of only 28 bp—to be imported into the genome during natural transformation.

## COMMENTARY

Helicobacter pylori lives in the human stomach. Rather than being digested, it survives and thrives by living in a kind of demimonde, the mucus layer between the epithelial cells and the stomach interior. Too close to the epithelium, and it must deal with the full force of the human immune system. Too far away, and it will eventually be overcome by the acidity of the stomach lumen. Nevertheless, it can survive decades in a single host by manipulating the behavior of human cells (1), both triggering the immune response and regulating its local expression (2), allowing it to carve out stable bolt holes within the mucus layer. Eventually, in pathogenic cases, persistent inflammation during decades of infection causes a cascade of changes in the gastric epithelia, leading eventually to cancer (3).

H. pylori is also remarkable because of its peculiar transmission genetics. It has a higher mutation rate than other bacteria, with around 30 mutations per genome per year, rather than 1 or 2, which is typical in other species (4). More striking still is the prodigious rate at which it exchanges DNA with other H. pylori strains. Mixed infection of the stomach by multiple strains is common, especially in the developing world, and cohabiting strains can exchange 10% or more of their DNA (5, 6). The rate of genetic exchange is highest in immune-related genes (7), presumably prolonging infections by allowing strains to escape from the evolving immune response.

The consequence of high rates of genetic exchange is that at a population level, there is the closest approximation to random assortment of polymorphisms found in any organism, or “free recombination” (8). The effect of recombination is quantified using linkage disequilibrium (LD) statistics (LD), which are calculated between pairs of polymorphisms (9). LD is highest when measured between sites next to each other in the genome and falls off progressively as a function of chromosomal distance. According to LD measures, H. pylori is a clear outlier, with LD between sites in the genome 10 bases apart being less than LD found in other bacteria at 100 bases (10). In other words, if you follow the history of a segment of DNA as it is transmitted from generation to generation, with both mutations and recombination events, splicing together DNA from different strains happens much more frequently in H. pylori than for any other organism that has been investigated.

Is the enormous flux of DNA experienced by the H. pylori genome an adaptation to its unusual niche, or is it the consequence of a historical accident? H. pylori lacks many homologues of the genes encoding DNA transmission and repair machinery that are found in Escherichia coli and lacks a conventional mismatch repair system, making it a “natural mutator” (11). One possible scenario is that the first H. pylori strain that colonized humans was deficient in the normal machinery used to transmit DNA faithfully. Since the bacterium was alone in its new niche, it was unable to repair its machinery by importing functional genes from related organisms. As a result of this historical accident, the genome found itself stuck in a kind of genetic purgatory, avoiding complete mutational meltdown only by patching itself up continuously using DNA taken from other strains. In fact, despite this apparent handicap the species has survived for a prolonged period, accompanying humans for at least the last 100,000 years (12), perhaps because H. pylori is protected from competition with organisms with more efficient genetic transmission mechanisms by its special, difficult-to-invade niche.

Arguments that the transmission genetics of H. pylori might be, at least in part, adaptive come from investigating the replication and recombination machinery that H. pylori does have. *In vitro* experiments in which H. pylori strains were exposed to naked DNA from other strains before having their genomes sequenced found that homologous recombination following transformation introduced “macroimports,” segments of DNA with an average length of 1,645 bp into the genome (13). It is difficult to explain the rapid breakdown of LD as a function of distance observed in H. pylori based on import of long segments of DNA because LD is only broken down when there is a recombination boundary between sites. Bubendorfer et al. (13) also observed “microimports” with a mean length of 28 nucleotides, which can potentially be more efficient in breaking down short-range LD between markers. Ailloud et al. (11) now demonstrated that these microimports arise when DNA from the donor strain is chopped into bits and interspersed with homologous DNA from the recipient strain. The shorter tracts frequently clustered together with other imported stretches, suggesting that their import is precipitated by a single DNA molecule entering the cell.

Ailloud et al. also made significant progress in elucidating the mechanistic basis of the very short import events, by showing that the gene encoding the UvrC endonuclease is essential for them to occur (11). The established role of UvrC in E. coli is in helping to repair DNA damage in response to UV radiation, where it catalyzes incisions on both sides of the DNA lesion during the repair process. In H. pylori, UvrC also participates in the same repair pathway while also having this secondary role in facilitating import of very short DNA segments. Ailloud et al. mutated conserved domains in UvrC that have been shown to be essential for generating incisions and found that this did not disrupt generation of short imports. Therefore, the mechanism of action in DNA damage repair and in facilitating recombination is independent of its catalytic activity and distinct from that underlying its role in DNA repair. Further experiments will be required to understand the mechanism in more detail.

It would be tempting to argue that the evolution of a specific mechanism for importing short segments implies that H. pylori has evolved specifically to reduce the LD in its genome and thereby to increase the efficiency of natural selection (14). However, there are caveats that make this conclusion premature. First, to demonstrate such hypotheses, we need to know when the novel features of the H. pylori recombination machinery evolved. Unfortunately, the origin of H. pylori is unknown. The closest relative, Helicobacter acinonychis, is found in big cats, but this seems to have been a human-to-cat host jump, rather than the other way around (15). Moreover, while there is evidence of frequent recombination within nonhuman *Helicobacter* (16), we do not have extensive samples from natural populations and therefore cannot easily compare recombination patterns. Comparative analysis of DNA repair systems is also challenging, especially because genes can be easily missed due to low homology with other better-studied bacteria (17). Second, the frequency of the short imports observed in Ailloud et al.’s experiments was low, corresponding to a few percent of the overall genetic exchange observed. This means that their overall role in reducing LD is likely modest unless such events occur at higher rates in natural genetic exchange events in the human stomach. Third, although breaking up LD generates new combinations of alleles that can be adaptive, this recombination also imposes a cost by breaking up existing favorable combinations and by introducing new mutations if the process goes wrong. The results of Ailloud et al. take us a step closer to understanding how H. pylori achieves free recombination. We still know little about why it does so.
